# Intensified therapy in diffuse large B-cell lymphoma

**DOI:** 10.18632/oncotarget.23770

**Published:** 2017-12-29

**Authors:** Mattia Novo, Alessia Castellino, Annalisa Chiappella

**Affiliations:** Hematology, Città della Salute e della Scienza Hospital and University, Torino, Italy

**Keywords:** diffuse large B-cell lymphoma, poor prognosis, high risk, high-dose chemotherapy, autologous stem cell transplant

Diffuse large B-cell lymphoma (DLBCL) is an heterogeneous disease; the standard treatment for DLBCL at diagnosis is a chemo-immunotherapy based on rituximab, cyclophosphamide, vincristine, doxorubicin, and prednisone performed every 21 days (R-CHOP21) [1], but 30-40% of patients still relapse. In young DLBCL patients with favorable prognosis according to the International Prognostic Index (IPI), the long-term cure rate after R-CHOP exceeds 80% [2], but in patients at high and high-intermediate IPI risk score, the prognosis still remains unsatisfactory. In the rituximab era, in order to improve the outcome of poor prognosis DLBCL, some strategies have been investigated, such as the dose-dense chemo-immunotherapy, based on the concept of reduce the time between two consequent cycles in order to increase the dose-intensity of the drugs; the results of a randomized phase III trial showed that the dose-dense R-CHOP14 was superimposable to standard R-CHOP21 in terms of progression free survival (PFS) and overall survival (OS) [3]. Another approach was to test the benefit of rituximab in addition to high-dose chemotherapy followed by autologous stem cell transplantation (R-HDC+ASCT) as first-line treatment; this strategy has been for long time matter of debate. Recently, four randomized trials conducted by collaborative international groups were published. Stiff, et al [4], in the SWOG-9704 trial, randomized patients affected by aggressive lymphomas, diagnosed between 1999 to 2007, in remission after five cycles of CHOP21 (with or without rituximab) to receive three more cycles of CHOP21 or one cycle of CHOP21 followed by ASCT conditioned by total body irradiation or carmustine containing regimen. The study showed an improvement in PFS in transplantation group compared with no transplantation, but no benefit in OS was observed.

A benefit in terms of Failure Free Survival (FFS) was reported also by Chiappella, et al [5], in the phase 3 randomized trial FIL-DLCL04 conducted by Fondazione Italiana Linfomi. In the Italian trial, 399 young patients with DLBCL at high-risk (age-adjusted IPI score 2 or 3) diagnosed between 2006 and 2010, were randomized to receive a short course of dose-dense chemo-immunotherapy at two different level of intensification (RCHOP/RMega-CHOP) followed by an intensive consolidation based on high-dose cytarabine, mitoxantrone and dexamethasone, followed by ASCT conditioned by BEAM regimen (carmustine, cytarabine, etoposide, melphalan), or a full course of dose-dense chemo-immunotherapy as part of first-line treatment. The study met the primary endpoint, demonstrating an advantage in FFS at 2-year of 71% (95%, CI 64–77) in the transplantation group versus 62% (95%, CI 55–68) in the non-transplant group, hazard ratio (HR) 0.65 (95%, CI 0.47–0.91), but no difference in OS was reported: 78% (95% CI 71–83) versus 77% (71–83), respectively; HR 0.98 (0.65–1.48).

No differences neither in OS nor in PFS between transplant and no transplant were reported in other two studies, the DSHNHL 2002-1 and the GITIL trial. The DSHNHL 2002-1 trial, designed by the German High-Grade Lymphoma Study Group, compared dose-dense R-CHOP plus etoposide regimen (R-CHOEP-14) and maximally dose-escalated R-MegaCHOEP followed by ASCT in a population of DLBCL diagnosed between 2003 to 2009. The R-MegaCHOEP transplantation approach was substantially more toxic, but no gain to no transplantation was seen [6]. Not even the study GITIL trial, comparing eight cycles of R-CHOP14 with an high-dose sequential chemotherapy program, determined significant differences in outcome between groups [7].

The absence of advantage in term of overall survival, may be explained by the use of R-HDC+ASCT as standard and effective strategy in young DLBCL patients in first relapse; hence, actually there are no data that justify the intensification with autologous stem cell transplantation as standard treatment in high-risk DLBCL at diagnosis.

Since the pre-rituximab era, a high-dose cisplatin and citarabine cointaing regimen (DHAP) followed by HDC and ASCT was considered the standard treatment in relapsed/refractory DLBCL patients, with a 5-years OS of 53%. In young patients eligible to intensive treatment, in relapsed and refractory setting, the HDC+ASCT is still the standard of care. The addition of rituximab to second-line chemotherapy followed by ASCT significantly improved PFS in patients not exposed to rituximab as part of their first-line treatment. In the CORAL trial [8], prior rituximab treatment, early relapse (within 12 months) and an high score age-adjusted IPI score, were identified as prognostic parameters at relapse correlated to lower 3-years PFS and OS (p < .001). In particular, early relapse and prior rituximab exposure, identified a group at dismal prognosis, with 3-year PFS of 23%. However, even in this unfavorable population, patients who underwent ASCT showed a better outcome compared to those not underwent ASCT (3-year PFS of 39% vs 14%, p < .001). (Table 1).

**Table 1:** Comparison of trials investigating role of high-dose chemotherapy (HDC) and autologous stem cell transplantation (ASCT) in first-line therapy and efficacy of different regimens plus ASCT as salvage therapy

	Chiappella, et al, 2017 [5]	Cortellazzo, et al. 2016 [7]	Stiff, et al. 2013 [4]	Schmitz, et al. 2012 [6]	Gisselbrecht, et al. 2010 [8]
Previous treatment	none	none	none	none	R/R to first-line therapy
Median age (years)	49	51	51	49	54
N° of pts in transplant/no transplant group	199/200	120/126	125/128	130/132	396/−
Progression free survival/Failure free survival (all patients)	71% vs 62% (2-yrs FFS)	75% vs 65% (3-yrs PFS)	69% vs 55% (2-yrs PFS)	70% vs 74% (3-yrs PFS)	37% (3-yrs PFS)*
Overall survival (all patients)	66% vs 67% 5-yrs OS)	77% vs 74% (3-yrs OS)	74% vs 71% (2-yrs OS)	77% vs 85% (3-yrs OS)	49% (3-yrs OS)*
Progression free survival (aa-IPI 2)	75% vs 65%	80% vs 64%	66% vs 63%	63% vs 75%	-
Overall survival (aa-IPI 2)	81% vs 81%	85% vs 81%	70% vs 75%	77% vs 91%	-
Progression free survival (aa-IPI 3)	62% vs 53%	67% vs 55%	75% vs 41%	55% vs 54%	-
Overall survival (aa-IPI 3)	69% vs 68%	67% vs 65%	82% vs 64%	77% vs 68%	-

aa-IPI: age-adjusted International Prognostic Index; PFS: progression free survival; FFS: failure free survival; OS: overall survival.

^*^ No difference between Rituximab-Ifosfamide-Carboplatin-Etoposide and Rituximab-Cisplatin-Citarabine-Dexametasone regimens.

Nevertheless, the treatment of poor prognosis DLBCL remain an unmet clinical need. A better knowledge of biological characteristics of the disease, should identify subgroup of DLBCL at different prognosis. On the bases of immuno-histochemistry, citogenetics and gene expression profiling, it is nowadays possible to identify different aggressive lymphoma, such as activated-B-cell lymphoma, double-expressor and double-hit. Novel drugs, such as lenalidomide and ibrutinib, showed to be able to overcome chemo-immunotherapy resistence in relapse and refractory DLBCL, and are under investigation in addition to standard chemo-immunotherapy in first line setting.
